# The predictive value of NLR and PLR for TACE prognosis in hepatocellular carcinoma: a systematic review and meta-analysis

**DOI:** 10.1515/med-2026-1378

**Published:** 2026-05-07

**Authors:** Chuanbao Jing, Zhijun Zhao, Ying Xin, Jiaye Long

**Affiliations:** Department of Urology and Vascular Surgery, Fourth People’s Hospital of Hulunbuir City, Hailar, China; Department of General Surgery, Inner Mongolia Forestry General Hospital, Second Affiliated Hospital of Inner Mongolia University for Nationalities, Yakeshi, China; Integrated TCM & Western Medicine Department, Fourth People’s Hospital of Hulunbuir City, Hailar, China; Hematology and Intervention Department, Xinhui Hospital Affiliated to Southern Medical University, Xinhui District People’s Hospital, Jiangmen, China

**Keywords:** HCC; TACE; NLR, PLR

## Abstract

**Introduction:**

The purpose of this study was to evaluate the predictive value of the neutrophil-to-lymphocyte ratio (NLR) and platelet-to-lymphocyte ratio (PLR) for outcomes in patients with hepatocellular carcinoma (HCC) undergoing transarterial chemoembolization (TACE).

**Content:**

We conducted a systematic search on PubMed, Embase, the Cochrane Library, and Web of Science, with a deadline of June 15, 2025. The statistical analysis of this study was conducted using R software (version 4.3.1). This meta-analysis was reported in accordance with the PRISMA guidelines. The research has been registered in PROSPERO (CRD42022361556).

**Summary:**

A total of 24 studies, involving 9,257 patients, were included. Our study suggested that elevated NLR values were associated with poorer overall survival (OS) (hazard ratio [HR]=1.73; 95 % confidence interval [CI]: 1.42–2.10) and progression-free survival (PFS) (HR=1.44, 95 % CI: 1.28–1.63) in patients. However, there was no statistically significant difference between the elevated PLR values and patient’s OS (HR=1.26, 95 % CI: 0.92–1.73) and PFS (HR=1.86, 95 % CI: 0.45–7.78).

**Outlook:**

Our meta-analysis suggested that high NLR before TACE was a predictor of poor OS and PFS prognosis in HCC patients after TACE. However, the prognostic value of PLR was not statistically significant and further prospective studies were needed to validate them.

## Introduction

Hepatocellular carcinoma (HCC) ranks as the sixth most common cancer and the second leading cause of cancer-related deaths globally [1]. While therapeutic procedures like surgical resection may be appropriate for early-stage HCC patients, the majority are diagnosed in intermediate to advanced stages, thus losing the opportunity for surgical intervention [2]. For patients unable to undergo surgical resection, transarterial chemoembolization (TACE) has emerged as the primary treatment, particularly for those in the middle stage [3]. TACE causes tumor necrosis by selective arterial embolization while providing localized chemotherapy, resulting in dual ischemic and cytotoxic effects. Nonetheless, substantial disparities exist in the therapeutic outcomes of TACE [4]. Consequently, identifying dependable prognostic biomarkers to assess the effectiveness of TACE is presently paramount.

Recent evidence has shown that systemic inflammation plays a significant role in HCC’s pathogenesis, progression, and prognosis [5]. Inflammatory cells and their mediators in the tumor microenvironment are key players in immune evasion. Furthermore, they enhance angiogenesis, invasion, and metastasis, thereby contributing to the aggressive nature of HCC. Inflammatory biomarkers, such as neutrophil-to-lymphocyte ratio (NLR) and platelet-to-lymphocyte ratio (PLR), have gained prominence in cancer prognosis research due to their affordability and accessibility [6]. The determination of NLR relies on the absolute numbers of peripheral blood neutrophils and lymphocytes. An elevation in NLR typically signifies an augmented pro-inflammatory milieu driven by neutrophils, whilst a reduction in lymphocytes may imply a suppression of anti-tumor immune activity. Likewise, PLR, which is derived from platelet and lymphocyte counts, suggests that a high ratio reflects platelet-mediated metastasis and angiogenesis, as well as diminished immune surveillance.

Numerous clinical investigations have shown that increased pre-treatment NLR and PLR levels are significantly associated with negative outcomes in HCC patients [7], 8]. Patients with high NLR or PLR often had lower tumor response rates, shorter progression-free survival (PFS), and inferior overall survival (OS) after TACE [7], 8]. The fundamental processes that connect these inflammatory indicators to TACE efficacy involve several routes. Initially, neutrophils help develop a pro-tumorigenic milieu by producing reactive oxygen species (ROS) and matrix metalloproteinases (MMPs), which increase tumor invasiveness and fibrosis [9]. Furthermore, decreased lymphocyte levels impair antitumor immunity, raising the likelihood of recurrence and metastasis. Likewise, platelets promote tumor vascularization and metastatic potential by secreting pro-angiogenic substances like vascular endothelial growth factor (VEGF) and platelet-derived growth factor (PDGF) [10]. These results suggest that NLR and PLR are not only reliable prognostic indicators, but also have the potential to predict the response to TACE treatment, which could be beneficial in patient stratification.

​The prognostic significance of NLR and PLR in patients receiving TACE remains contentious. Some studies have indicated that they serve as independent prognostic variables and have suggested various cut-off values [11]. However, other studies indicate that these cut-off levels may be constrained by confounding variables such as tumor burden, liver function status, and etiology [12], 13]. Hence, a meta-analysis is necessary to assess the predictive significance of NLR and PLR in TACE treatment.

## Methods

### Search strategy

The PRISMA guidelines were strictly followed in this study [14]. A full systematic search was carried out across PubMed, Embase, the Cochrane Library, and Web of Science from inception to June 15, 2025. The search strategy employed the following keywords and their combinations: (“Transarterial chemoembolization “ OR “TACE”) AND (“Neutrophil-to-lymphocyte ratio” OR “NLR” OR “Platelet-to-lymphocyte ratio” OR “PLR”). For detailed search strategies, please refer to the Supplementary materials 1. This study was prospectively registered with PROSPERO (CRD420251078905).

### Selection criteria

This study employed the following screening criteria: (P) Patients with HCC confirmed by histopathology or imaging who underwent TACE; (I) pre-treatment NLR/PLR measurement; (C) studies providing defined NLR/PLR cut-off values with low/high-level stratification; (O) reported OS/PFS outcomes with hazard ratio (HR) and 95 % confidence interval (CI); (S) prospective/retrospective cohort studies.

The exclusion criteria included: (1) receipt of any non-TACE therapy either before or during TACE treatment follow-up; (2) case reports or review articles.

The literature screening was independently completed by two researchers (L.J.Y and J.C.B). A preliminary screening was conducted by reading the titles and abstracts, followed by a full-text evaluation of potentially eligible literature. Differences were resolved through discussion or consultation with a third researcher (Z.Z.J).

### Data extraction

The following data were systematically extracted from each included study: first author, publication year, country/region of origin, sample size, mean/median patient age, follow-up duration, predefined NLR/PLR cut-off values, Child-Pugh class, BCLC stage classification, as well as HR and their 95 % CI for survival outcomes. For the extraction of HR and their standard errors (SEs), the HR values were obtained directly when available, accompanied by their 95 % CIs. The SEs were subsequently calculated using the formula: SE=(log (upper CI) – log (lower CI))/(2 × 1.96). The Kaplan-Meier plots were used to extract data for studies that did not provide HR values using Engauge Digitizer version 11.3 software.

### Quality assessment

Study quality was assessed using the Newcastle-Ottawa Scale (NOS) [15], which evaluated three domains: (1) participant selection (0–4 points), (2) between-group comparability (0–2 points), and (3) outcome assessment (0–3 points). Based on total scores (maximum 9 points), studies were categorized as high-quality (7–9 points), moderate-quality (5–6 points), or low-quality (≤4 points).

### Statistical analysis

The primary analysis calculated pooled HR with 95 % CI to assess the prognostic value of baseline NLR and PLR. Heterogeneity was evaluated using I^2^ statistics and Cochran’s Q test, with I^2^ >50 % or Q-test p<0.10 indicating substantial heterogeneity and warranting a random-effects model. Otherwise, a fixed-effects model was applied. Subgroup analyses stratified by study region, sample size, and NLR/PLR cut-off values were conducted to explore heterogeneity sources. In addition, we also conducted meta-regression to evaluate whether the heterogeneity between studies was attributed to covariates. We employed the leave-one-out method, systematically removing one study at a time to assess its influence on the overall pooled effect. Publication bias was assessed via Egger’s regression test and funnel plot symmetry. A two-sided p-value <0.05 was considered statistically significant. All statistical analyses were performed using R software (version 4.3.1). The R software packages packages used in this study include “meta”, “ggplot2”, “readxl”, and “robvis”.

## Results

### Literature search

**Figure 1:**
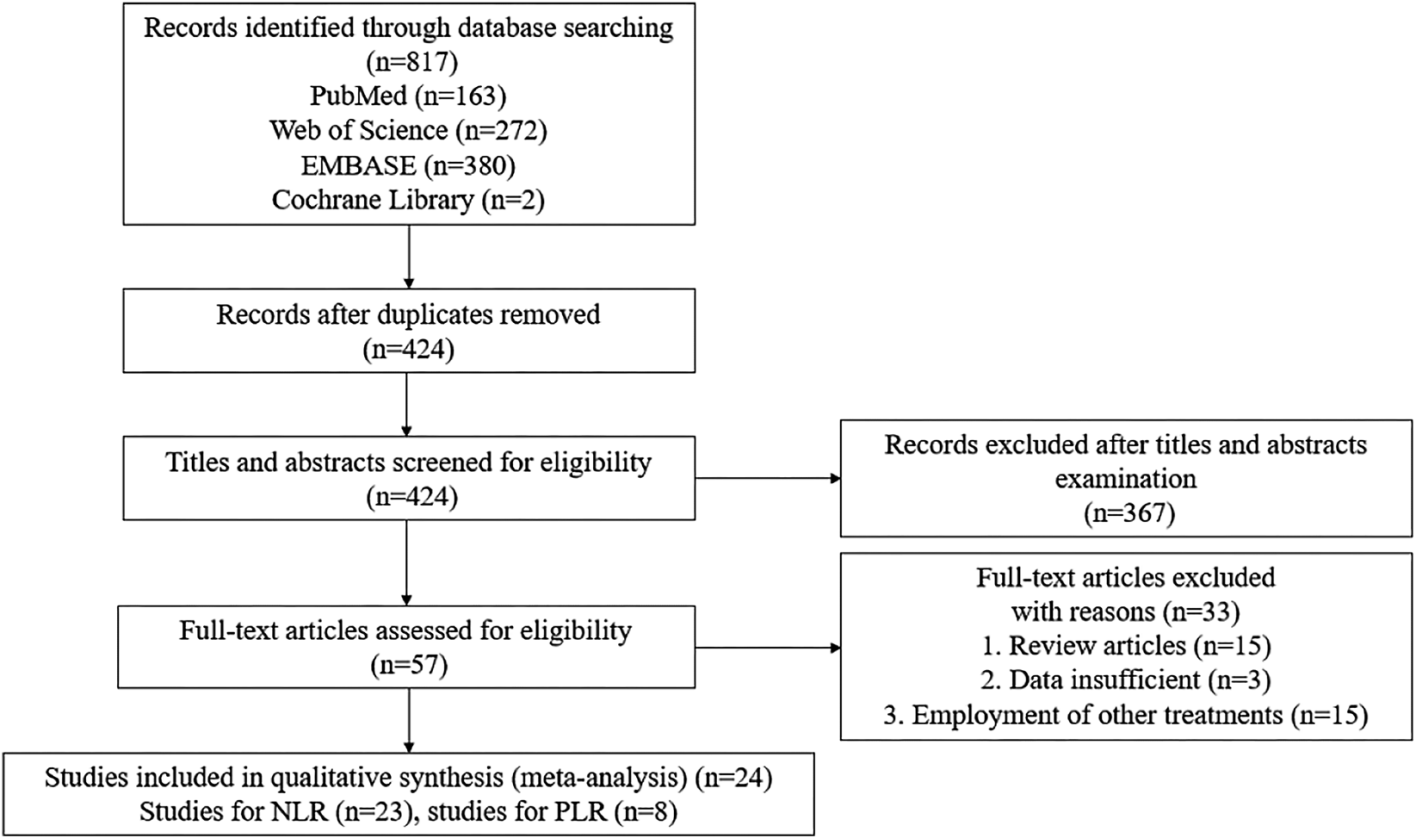
Literature search and screening process.

A total of 817 records were identified from 4 databases. Following removal of 393 duplicate records, 424 unique records were subjected to title and abstract screening. This screening led to the exclusion of 367 unrelated studies. Subsequently, a further 33 articles were excluded: 15 review articles, 3 articles with insufficient data, and 15 articles investigating other interventions or treatments. Consequently, 24 articles were included in the meta-analysis, of which 23 investigated the NLR and 8 examined the PLR (Figure 1).

### Study characteristics

There were a total of 9,257 patients in the 24 studies. Individual study sample sizes ranged from 54 to 1,625 patients. Geographically, 16 studies originated from China [7], 12], 13], 16], [19], [20], [21], [22], [23], [24], [25, [28], [29], [30, 34], 35], 3 from Korea [27], 31], 33], 3 from USA [8], 18], 32], and 2 from Italy [17], 26]. Twenty studies investigated the association between pre-TACE NLR values and OS [12], 13], [16], [17], [18], [19], [20, [22], [23], [24], [25], [26], [27], [28], [29], [30], [31], [32], [33], [34], while six studies examined pre-TACE PLR and OS [12], 13], 21], 22], 28], 32]. Pre-TACE NLR in relation to PFS was reported in five studies [7], 8], 26], 32], 35], and pre-TACE PLR with PFS was reported in three studies [7], 8], 32]. Across the studies, the cut-off values varied from 1.57 to 5.0 for NLR and from 63.5 to 150 for PLR. The average score of NOS was 7.8 (SD=0.96) (Table 1 and Supplementary materials 2). The study characteristics are detailed in Table 1.

**Table 1:** Characteristics included in the study.

Author	Country	Sample size (n, male)	Mean/median ages, years	Child-pugh class	BCLC stage	Follow-up time, months	Cut-off value	Outcome	NOS score
Huang (2011) [16]	China	145 (134)	49	A	NA	24	NLR=3.3	OS	9
Pianto (2012) [17]	Italy	54 (40)	63	A/B	A/B/C	82	NLR=5	OS	6
McNally (2013) [18]	USA	104 (77)	56	A/B	NA	50	NLR=5	OS	8
Xu (2014) [19]	China	178 (149)	54.3	A/B	B	110	NLR=1.85	OS	7
Zhang (2014) [20]	China	138 (99)	56.8	A/B	NA	120	NLR=5	OS	7
Xue (2015) [21]	China	291 (258)	53.05	A/B	B/C	50	PLR=150	OS	9
Tian (2016) [22]	China	122 (107)	56	A/B	NA	94	NLR=2.61 PLR=96.13	OS	7
Zhou (2016) [23]	China	279 (251)	50	A/B	NA	26	NLR=2.6	OS	8
Liu1 (2017) [24]	China	793 (665)	54	A/B	B/C	83	NLR=2.2	OS	7
Liu2 (2017) [25]	China	760 (643)	56.5	A/B	NA	83	NLR=2.2	OS	8
Rebonato (2017) [26]	Italy	49 (39)	75	A/B	B/C	47	NLR=2.03	OS PFS	9
Chon (2019) [27]	Korea	921 (700)	68.2	A/B	A/B/C	108	NLR=5	OS	9
He (2019) [28]	China	216 (200)	60	A/B	A/B/C	96	NLR=1.77 PLR=94.62	OS	9
Liu (2020) [29]	China	180 (155)	54.3	A/B	B/C	64	NLR=3.94	OS	9
Schobert (2020) [8]	USA	46 (37)	64.44	A/B/C	A/B/C	72	NLR=3.22 PLR=113.1	PFS	7
Wang (2020) [30]	China	380 (311)	54.3	A/B	A/B/C	65	NLR=2.4	OS	8
Chu (2021) [31]	Korea	1,121 (810)	59.4	A/B	B	108	NLR=3	OS	9
Liu (2021) [7]	China	128 (112)	56.96	A/B	B	65	NLR=1.57 PLR=92	PFS	7
Lu (2021) [12]	China	1,625 (1,471)	60	NA	A/B/C	94	NLR=5 PLR=150	OS	8
Young (2021) [32]	USA	296 (232)	61.4	NA	NA	90	NLR=2.7 PLR=88.3	OS PFS	8
Cho (2022) [33]	Korea	605 (497)	57	A/B	A/B/C	23	NLR=1.7	OS	7
Wang (2023) [34]	China	364 (300)	58	A/B	A/B/C	78	NLR=4	OS	9
Xi (2023) [35]	China	346 (306)	53	NA	A/B/C	36	NLR=4.4	PFS	7
Zhou (2024) [13]	China	116 (104)	68.5	NA	NA	43	NLR=3.465 PLR=63.5	OS	6

BCLC, Barcelona clinic liver cancer; NLR, neutrophil-to-lymphocyte ratio; PLR, platelet-to-lymphocyte ratio; OS, overall survival; PFS, progression-free survival; NOS, the newcastle-ottawa scale.

### Relationship between pre-TACE NLR/PLR values and OS/PFS in HCC patients

**Figure 2:**
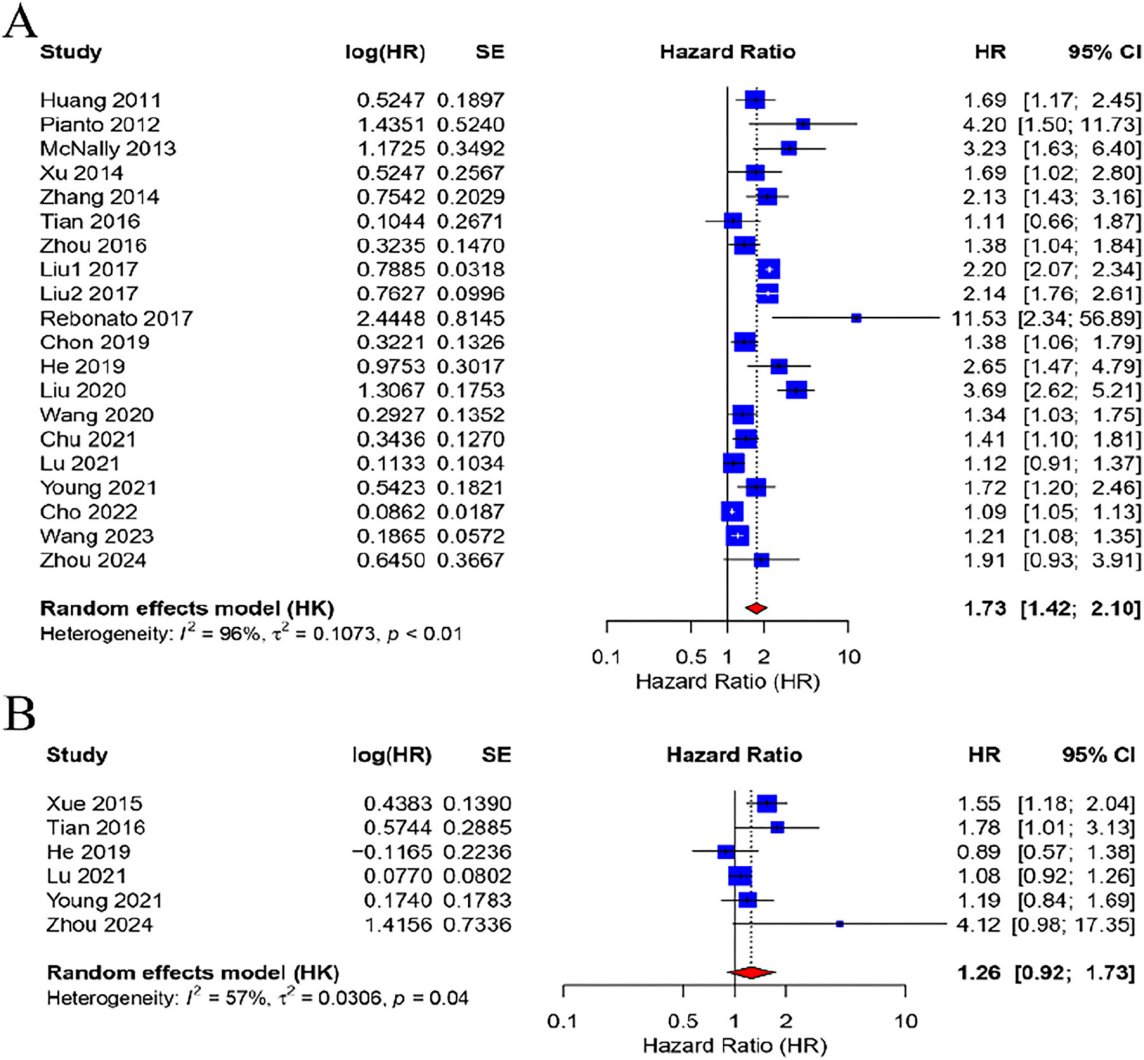
Forest plot of the relationship between NLR/PLR values before TACE and patient OS. Each blue square in the graph corresponds to a separate study, and the size of the square reflects its relative weight in the analysis. The horizontal line passing through each square represents the 95 % CI of the observed effect. At the bottom of the graph, the red diamond represents the combined effect calculated in all included studies, and the width of the diamond represents the 95 % CI. (A) NLR-OS; (B) PLR-OS. HR, hazard ratio; CI, confidence interval; SE, standard error; NLR, neutrophil-to-lymphocyte ratio; PLR, platelet-to-lymphocyte ratio; TACE, transarterial chemoembolization; OS, overall survival.

**Figure 3:**
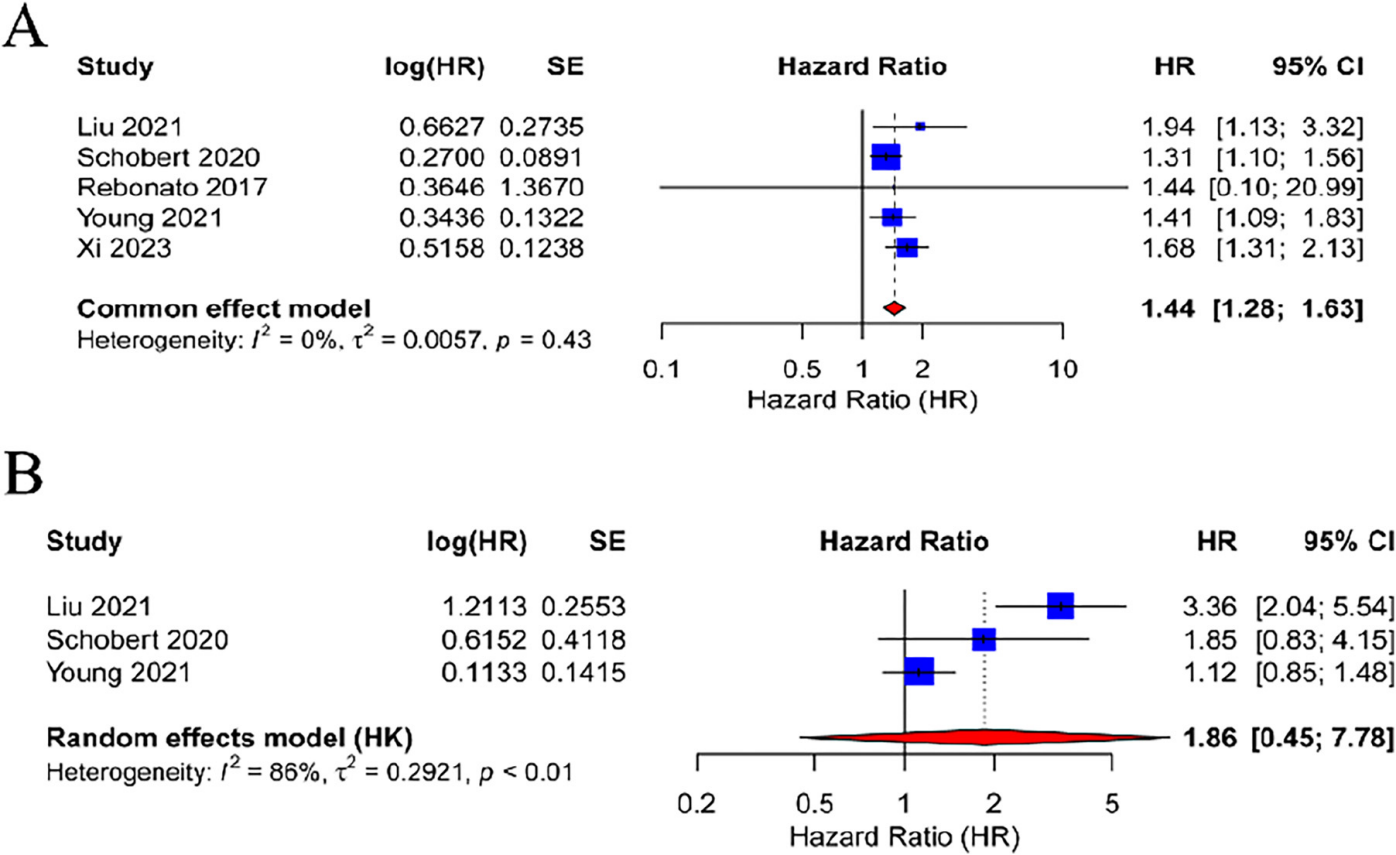
Forest plot of the relationship between NLR/PLR values before TACE and patient PFS. Each blue square in the graph corresponds to a separate study, and the size of the square reflects its relative weight in the analysis. The horizontal line passing through each square represents the 95 % CI of the observed effect. At the bottom of the graph, the red diamond represents the combined effect calculated in all included studies, and the width of the diamond represents the 95 % CI. (A)NLR-PFS; (B) PLR-PFS. HR, hazard ratio; CI, confidence interval; SE, standard error; NLR, neutrophil-to-lymphocyte ratio; PLR, platelet-to-lymphocyte ratio; TACE, transarterial chemoembolization; PFS, progression-free survival.

In HCC patients, a pooled analysis of 20 studies using a random-effects model indicated that elevated pre-TACE NLR levels were significantly associated with OS, with an HR of 1.73 (95 % CI: 1.42–2.10; p<0.001), suggesting a 73 % increased risk of mortality. Notably, substantial heterogeneity was observed among these studies (I^2^=96 %, p<0.01) (Figure 2A). Conversely, the predictive value of pre-TACE PLR for OS was assessed in six studies, revealing no statistically significant association between high PLR and OS, with moderate heterogeneity (I^2^=57 %, p=0.04) and an HR of 1.26 (95 % CI: 0.92–1.73; p=0.15) (Figure 2B). A fixed-effects model was employed for the analysis due to the low heterogeneity among studies (I^2^=0 %, p=0.43), revealing that elevated NLR significantly predicted worse PFS, with an HR of 1.44 (95 % CI: 1.28–1.63; p<0.001), indicating a 44 % increased risk of disease progression (Figure 3A). In contrast, three studies assessing pre-TACE PLR and PFS using a random-effects model (I^2^=86 %, p<0.01) revealed no significant association (HR=1.86, 95 % CI: 0.45–7.78; p=0.39) (Figure 3B).

### Subgroup analysis and meta-regression

The results of the subgroup analyses are summarized in Tables 2 and 3.

**Table 2:** Subgroup analysis of the prognostic value of NLR and PLR for OS after TACE in HCC patients.

Subgroups		Included studies	HR	95 % CI	I^2^	p-Value
NLR	Overall	20	1.73	1.42–2.10	96 %	<0.01
Region	Eastern	16	1.63	1.35–1.96	97 %	<0.01
	Western	4	3.15	1.04–9.52	65 %	0.03
Sample size	≤150	7	2.15	1.27–3.63	58 %	0.03
	>150	13	1.62	1.30–2.00	97 %	<0.01
Cut-off value	NLR ≤2.5	7	1.82	1.17–2.84	99 %	<0.01
	NLR >2.5	13	1.68	1.32–2.16	80 %	<0.01
PLR	Overall	6	1.26	0.92–1.73	57 %	0.04
Region	Eastern	5	1.30	0.82–2.06	65 %	0.02
	Western	1	1.19	0.84–1.69	NA	NA
Sample size	≤150	2	2.06	0.03–123.82	12 %	0.29
	>150	4	1.18	0.83–1.66	54 %	0.09
Cut-off value	PLR ≤100	4	1.30	0.63–2.68	54 %	0.09
	PLR >100	2	1.27	0.13–12.48	80 %	0.02

HR, hazard ratio; CI, confidence interval; SE, standard error; NLR, neutrophil-to-lymphocyte ratio; PLR, platelet-to-lymphocyte ratio; OS, overall survival; PFS, progression-free survival.

**Table 3:** Subgroup analysis of the prognostic value of NLR and PLR for PFS after TACE in HCC patients.

Subgroups		Included studies	HR	95 % CI	I^2^	p-Value
NLR	Overall	5	1.44	1.28–1.63	0 %	0.43
Region	Eastern	2	1.72	1.38–2.14	0 %	0.62
	Western	3	1.34	1.15–1.55	0 %	0.90
Sample size	≤150	3	1.46	1.05–2.02	0 %	0.39
	>150	2	1.55	1.29–1.84	0 %	0.34
Cut-off value	NLR ≤2.5	2	1.92	1.13–3.24	0 %	0.83
	NLR >2.5	3	1.43	1.24–1.66	23 %	0.27
PLR	Overall	3	1.86	0.45–7.78	86 %	<0.01
Region	Eastern	1	3.36	2.04–5.54	NA	NA
	Western	2	1.24	0.83–1.84	25	0.25
Sample size	≤150	2	2.72	1.56–4.76	34 %	0.22
	>150	1	1.86	0.94–3.70	NA	NA
Cut-off value	PLR ≤100	2	1.90	0.65–5.57	93 %	<0.01
	PLR >100	1	1.85	0.83–4.17	NA	NA

HR, hazard ratio; CI, confidence interval; SE, standard error; NLR, neutrophil-to-lymphocyte ratio; PLR, platelet-to-lymphocyte ratio; OS, overall survival; PFS, progression-free survival.

Subgroup analyses confirmed the prognostic value of pre-TACE NLR for OS across all stratifications. Regionally, Western countries exhibited a stronger association (HR=3.15, 95 % CI: 1.04–9.52) than Eastern countries (HR=1.63, 95 % CI: 1.35–1.96). Small-sample studies (≤150 patients) yielded a higher but less precise HR (HR=2.15, 95 % CI: 1.27–3.63) compared to large-sample studies (>150 patients; HR=1.62, 95 % CI: 1.30–2.00). All subgroups showed significant heterogeneity, suggesting that the prognostic value of NLR may be influenced by factors such as detection methods and patient characteristics.

No subgroup demonstrated statistically significant associations between pre-TACE PLR and OS. Eastern (HR=1.30, 95 % CI: 0.82–2.06) and Western (HR=1.19, 95 % CI: 0.84–1.69) cohorts showed comparable effects. Small-sample studies had volatile estimates (HR=2.06, 95 % CI: 0.03–123.82), while large-sample results were stable (HR=1.18, 95 % CI: 0.83–1.66). PLR cutoffs (≤100 vs. >100) similarly lacked significance. Moderate-to-high heterogeneity implied confounding by assay variability or clinical diversity.

NLR consistently predicted poorer PFS in all subgroups: Eastern (HR=1.72, 95 % CI: 1.38–2.14) and Western regions (HR=1.34, 95 % CI: 1.15–1.55), small (≤150 patients; HR=1.46) and large samples (>150 patients; HR=1.55), and across thresholds (≤2.5: HR=1.92; >2.5: HR=1.43). Low heterogeneity underscored robust inter-study consistency.

Subgroup analysis of pre-TACE PLR values for PFS in HCC patients revealed notable regional differences, with Eastern populations demonstrating a stronger association (HR=3.36, 95 % CI: 2.04–5.54) compared to Western cohorts (HR=1.24, 95 % CI: 0.83–1.84). The analysis showed a marked discrepancy between study sizes, with small-sample studies (HR=2.72, 95 % CI: 1.56–4.76) yielding substantially higher risk estimates than large-sample studies (HR=1.86, 95 % CI: 0.94–3.70). PLR cut-off analyses produced consistent but nonsignificant results (≤100: HR=1.90; >100: HR=1.85). Although some subgroups showed positive associations, due to high heterogeneity and insufficient sample size in some subgroups (such as only 2 studies in Western countries), current evidence could not establish PLR as an independent predictor of postoperative PFS after TACE.

Therefore, subgroup analysis showed that the value of NLR in predicting OS and PFS before treatment remained consistent across different regions, sample sizes, and threshold groups, suggesting its robustness as a prognostic marker. However, PLR did not demonstrate a stable or consistent predictive value for OS or PFS.

To further explore whether study-level characteristics could explain the substantial heterogeneity observed in the association between NLR/PLR and OS, we performed meta-regression including study region, sample size, and the NLR/PLR cut-off value as covariates (Table 4).

**Table 4:** Meta-regression analysis of the impact of different covariates on patient OS in NLR and PLR.

	Etimate	SE	zval	pval	Lower 95 % CI	Upper 95 % CI
NLR						
Intercept	0.82	0.70	1.20	0.23	−0.53	2.19
Region	0.53	0.28	1.87	0.06	−0.03	1.09
Sample size	−0.27	0.22	−1.23	0.22	−0.71	0.16
Cut-off value	−0.24	0.20	−1.21	0.23	−0.63	0.15
PLR						
Intercept	0.93	0.81	1.15	0.25	−0.66	2.52
Region	0.29	0.43	0.67	0.50	−0.55	1.14
Sample size	−0.85	0.46	−1.84	0.07	−1.75	0.06
Cut-off value	0.36	0.37	0.97	−0.33	−0.36	1.07

SE, standard error; CI, confidence interval; NLR, neutrophil-to-lymphocyte ratio; PLR, platelet-to-lymphocyte ratio.

For NLR, none of the covariates was significantly associated with the log (HR) for OS. Region showed a borderline association (p=0.06), whereas sample size (p=0.22) and NLR cut-off value (p=0.23) were not significant. A similar pattern was observed for PLR. None of the tested covariates reached statistical significance in the meta-regression (region: p=0.50; sample size: p=0.07; PLR cut-off: p=0.33).

### Sensitivity analysis and publication bias detection

**Figure 4:**
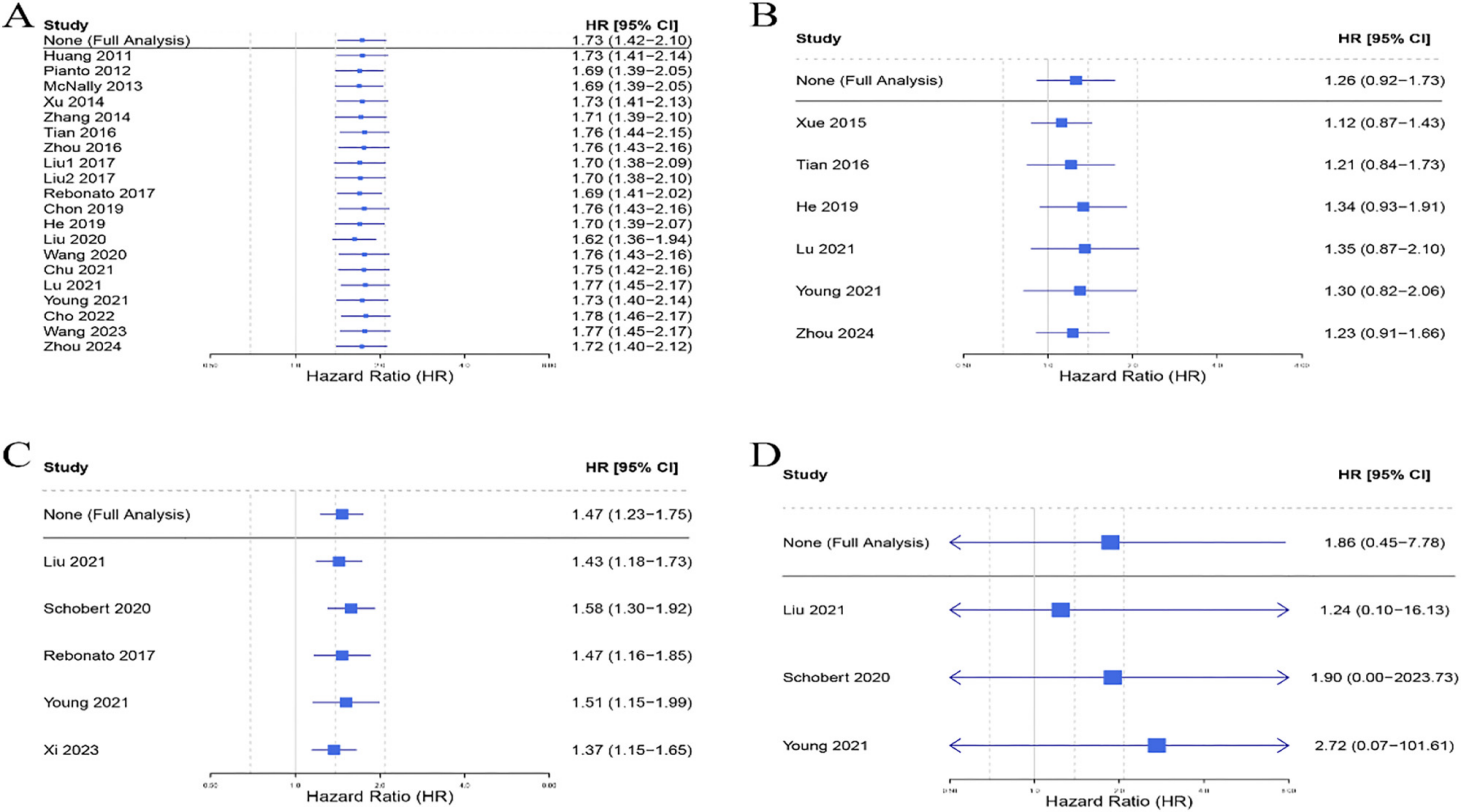
Sensitivity analysis of NLR and PLR in predicting TACE treatment OS and PFS. (A) NLR-OS; (B)PLR-OS; (C) PLR-PFS; (D) PLR-PFS. Each blue square in the graph corresponds to a separate study. The horizontal line passing through each square represents the 95 % CI of the observed effect. HR, hazard ratio; CI, confidence interval; SE, standard error; NLR, neutrophil-to-lymphocyte ratio; PLR, platelet-to-lymphocyte ratio; TACE, transarterial chemoembolization; OS, overall survival; PFS, progression-free survival.

Sequential exclusion of studies demonstrated stable pooled HR estimates across all analyses (Figure 4), confirming the robustness of our primary findings.

**Figure 5:**
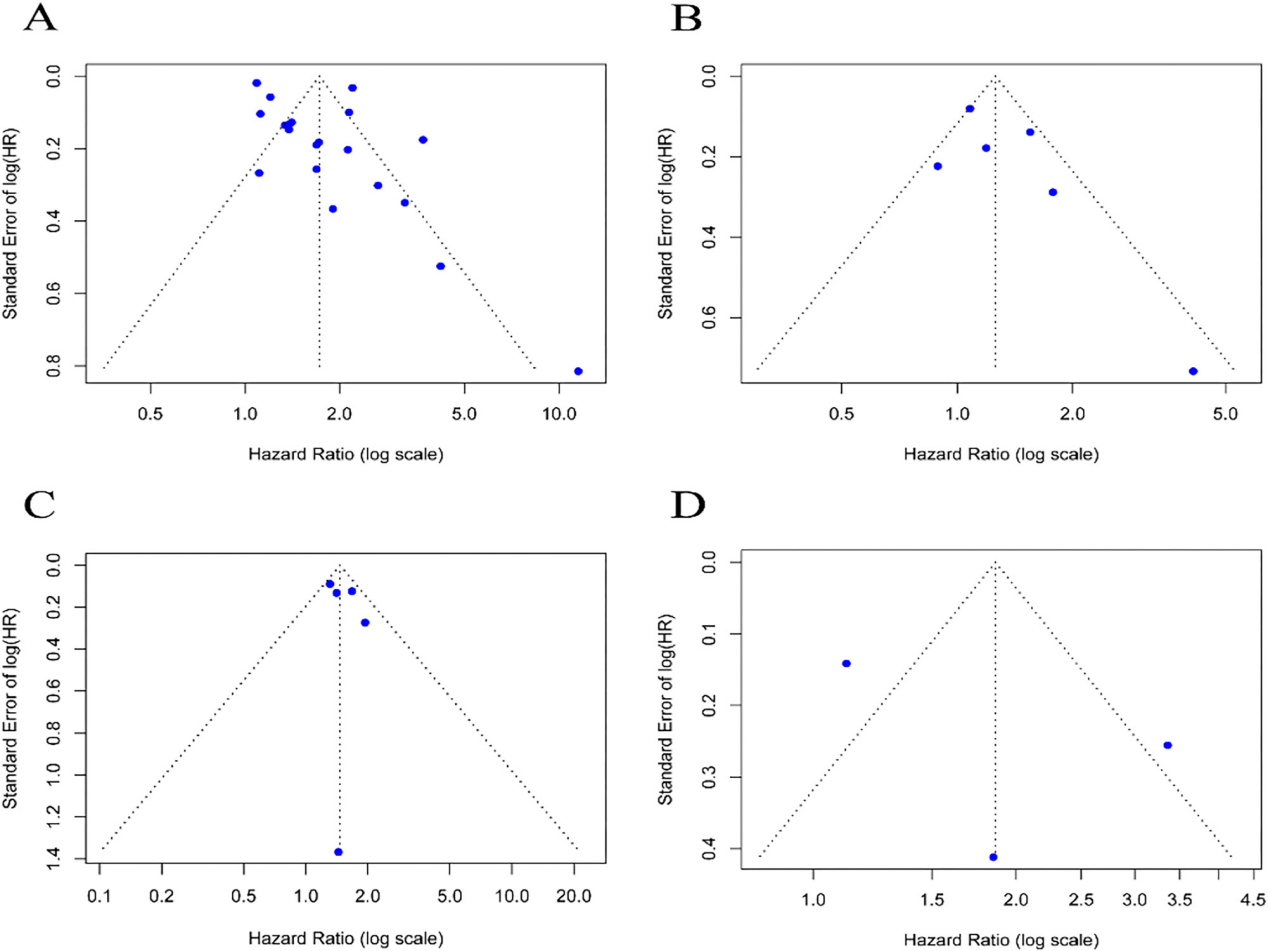
Funnel plot of NLR and PLR predicting OS and PFS of TACE treatment for HCC. (A) NLR-OS; (B) PLR-OS; (C) PLR-PFS; (D) PLR-PFS​. Circles, an individual study; diagonal lines, pseudo 95 % CI; middle vertical line, pooling HR. OS, overall survival; PFS, progression-free survival; HR, hazard ratio; CI, confidence interval.

Funnel plots assessing pre-TACE NLR and PLR effects on OS exhibited symmetrical point distributions about the midline (Figure 5A and B), suggesting no visual evidence of publication bias. This was supported by Egger’s test (NLR: p=0.100; PLR: p=0.133), which indicated no statistical evidence of publication bias. The funnel plots for PFS analyses similarly showed symmetrical distributions for both NLR (Figure 5C) and PLR (Figure 5D). While Egger’s test confirmed absence of bias for NLR (p=0.133), the test could not be performed for PLR due to insufficient included studies. Therefore, no significant publication bias was detected in this meta-analysis.

## Discussion

This meta-analysis, encompassing 24 studies with 9,257 HCC patients undergoing TACE, systematically evaluated the prognostic value of pre-treatment NLR and PLR. The results demonstrated that elevated NLR levels were significantly associated with worse OS (HR=1.73, 95 % CI:1.42–2.10) and PFS (HR=1.44, 95 % CI:1.28–1.63), with consistent findings across all subgroup analyses including geographical regions, study sizes, and cut-off thresholds. In contrast, PLR showed no statistically significant association with either OS (HR=1.26, 95 % CI:0.92–1.73) or PFS (HR=1.86, 95 % CI:0.45–7.78), with these analyses being limited by substantial heterogeneity and wide CI that precluded definitive conclusions. NLR may be clinically useful in risk stratification for HCC patients undergoing TACE, as evidenced by its strong and consistent prognostic value when compared to PLR.

The aforementioned results have clinical significance. The NLR measurement is cost-effective and easily calculable by standard blood testing, facilitating prognostic assessment of patients prior to TACE without incurring additional medical expenses. Secondly, owing to its prognostic prediction capability, it can help determine whether patients should receive TACE, particularly in economically disadvantaged regions. Ultimately, both OS and PFS associate with the NLR, enabling a preliminary assessment of patient survival and treatment response following TACE.

From a mechanistic point of view, the systemic inflammatory state shown by NLR is closely linked to several important steps of HCC progression. ROS and nitric oxide (NO) produced by neutrophils directly disrupt mitochondrial function in CD8+T cells, diminishing their glycolytic capacity and ultimately reducing the effector T cell population [36], 37]. Concurrently, lymphopenia not only serves as a marker of systemic immunosuppression but also facilitates tumor microenvironment remodeling through regulatory T cell (Treg) expansion [38], 39]. Our research results align with other prior studies. Liu et al. [29] believe that the high NLR group has a significantly shorter median OS than the low NLR group. Young et al. [32] also confirmed that NLR >2.7 is associated with shorter PFS. These findings are highly consistent with our merged results, strengthening the evidence base for NLR as a prognostic biomarker. Based on the above mechanisms and clinical evidence, NLR has clear clinical relevance and can be considered for integration into TACE treatment decision-making and risk stratification processes. Specifically, for patients with significantly elevated NLR before treatment, they can be identified as high-risk individuals and actively considered for combination systemic treatment strategies based on TACE therapy, such as combining targeted drugs or immune checkpoint inhibitors, to improve the immunosuppressive microenvironment and enhance anti-tumor efficacy. In addition, patients with high NLR may require closer postoperative follow-up and imaging evaluation to timely detect disease progression. Future research can further explore the inclusion of NLR as a supplementary evaluation indicator in the BCLC staging system, construct more personalized prognostic prediction models, and optimize patient stratification and management.

The predictive relevance of PLR is still debated in contemporary literature. Our meta-analysis failed to demonstrate statistically significant associations between PLR and either OS or PFS, a finding consistent with several previous reports. Lu et al. [12] reported no significant association between PLR and OS, while Young et al. [32] found only marginal predictive value for PFS. This inconsistency may stem from several factors: (1) Significant heterogeneity in patient populations across studies, particularly regarding baseline characteristics such as BCLC staging, tumor burden, liver function, and microvascular invasion status. For instance, while a retrospective study of 216 patients did show shorter OS in patients with PLR ≥94.62 (p=0.003), PLR failed to emerge as an independent prognostic factor in multivariate analysis when adjusted for NLR [28]. (2) The dual biological role of platelets in HCC pathogenesis–while promoting angiogenesis and metastasis, post-TACE thrombotic effects may paradoxically influence tumor blood supply, creating complex, non-linear relationships with survival outcomes that may obscure PLR’s predictive utility. These biological and methodological complexities underscore the need for well-designed prospective cohort studies to definitively evaluate pre-TACE PLR’s prognostic value in HCC patients. (3) The impact of research design and sample size. Because most of the studies included in this study were retrospective, limitations such as limited sample size, the retrospective nature of the data, and susceptibility to selective bias and information bias were present. When statistical power is insufficient, it is easy to draw inconsistent conclusions.

Our study found significant heterogeneity, particularly in the link between NLR and OS (I^2^=96 %). We found several potential sources of heterogeneity using subgroup analysis. First, there were regional disparities. The effect size reported in studies from Western countries was greater than in Eastern countries, which could be attributed to differences in demographic characteristics, etiologic composition, or medical procedures. For example, Western countries recorded HR levels as high as 4.20, whereas most Eastern countries had HR values ranging from 1.5 to 2.0. Second, there was a variation in sample sizes. Small sample studies had a higher effect value than large sample studies, indicating that they may be exaggerated. This tendency was most noticeable in Rebonato et al.’s study [26], which found an HR of 11.53. Third, the selection of cut-off values. The critical values of NLR ranged from 1.57 to 5.0, but curiously, the prediction directions of different critical value groups were consistent, with only minor changes in impact size, demonstrating that the predictive value of NLR had some tolerance for critical value selection.

However, when these study-level factors were formally evaluated using meta-regression, none of them showed a statistically significant modifying effect on the NLR–OS or PLR–OS association. For NLR, region, sample size and cut-off value all had p-values >0.05, although region showed a borderline trend (p=0.06). For PLR, region and cut-off value were not significant, and sample size showed only a borderline association (p=0.07). These findings indicate that the large between-study heterogeneity cannot be attributed to any single measured covariate, and that the subgroup differences should be considered exploratory and hypothesis-generating rather than definitive evidence of effect modification. The apparent discrepancy between subgroup analyses and meta-regression is not unexpected and has been reported in previous meta-analyses [40], 41]. Subgroup analyzes are prone to chance findings, especially when based on few studies per stratum, whereas meta-regression often has limited power to detect modest effect modification when the number of included studies is small and study-level covariates are associated. In our meta-analysis, most studies were conducted in Eastern populations, and several study characteristics, such as region, etiology, tumor stage, TACE protocols and cut-off selection were likely interrelated. Moreover, important clinical factors such as underlying liver function and detailed treatment regimens could not be consistently extracted, and therefore could not be examined as moderators.

It is worth noting that the NLR threshold range included in this study was 1.57–5.0, and the PLR threshold range was 63.5–150. This broad threshold difference may reflect differences in population characteristics, etiology of HCC, liver function status, tumor burden, and follow-up time, and may also be related to the testing units, laboratory methods used by each research institute, and whether interference factors such as infection have been excluded. Due to the fact that threshold differences do not alter the directionality of results, but may significantly affect the sensitivity and specificity of clinical stratification, it is not appropriate to directly generalize a single threshold to all clinical scenarios. In the future, we should explore establishing localized thresholds for specific populations or regions. Alternatively, a risk prediction model can be established in the form of continuous variables to predict the prognosis of HCC patients after receiving TACE.

This study focused on the predictive value of inflammatory markers before treatment for TACE prognosis in HCC patients, but has not yet included evidence of fertility preservation related interventions and their long-term outcomes. With the prolonged survival time of HCC patients, the combination of fertility desire and treatment decisions is increasingly attracting attention. The existing reviews and consensus documents have begun to focus on the feasibility and safety of controlled ovarian stimulation (COS) combined with oocyte/embryo freezing to preserve fertility in patients with HCC or other solid tumors [42], 43]. However, the evidence is still insufficient, and a systematic evaluation is needed based on factors such as liver function status, degree of liver cirrhosis, and treatment options. Future research should evaluate the safety, potential impact on tumor progression, and key endpoints of COS in HCC treatment populations within a strictly controlled design framework.

This study had several important limitations that should be considered. First, the predominance of retrospective study designs introduced potential selection and information biases that could affect the validity of our findings. Second, despite comprehensive subgroup analyses, substantial residual heterogeneity persisted, suggesting the influence of unaccounted confounding variables. Third, the limited number of studies examining PLR (n=6 for OS; n=3 for PFS) reduced statistical power and increased uncertainty in these estimates, particularly evidenced by the wide CI observed. Fourth, significant variability in follow-up duration across studies (range:23–120 months) may have led to incomplete endpoint ascertainment, particularly for long-term survival outcomes, potentially biasing the pooled estimates. Finally, although multiple sensitivity analyzes were conducted in this study, there are still inevitable sources of heterogeneity. Some data, such as baseline liver function, tumor burden, etiology, and TACE regimen, have not been fully reported in the existing literature. Future research should focus as much as possible on the above data as research directions, in order to provide more accurate explanations and quantitative analysis of heterogeneity.

## Conclusions

This meta-analysis indicates that high NLR before TACE is an effective predictor of poor OS and PFS prognosis in HCC patients after TACE, and has clinical applicability. However, the predictive value of PLR has not reached significance, and further research is needed to verify it in the future.

## Supplementary Material

Supplementary Material
