# Supplementary material for: The predictive value of NLR and PLR for TACE prognosis in hepatocellular carcinoma: a systematic review and meta-analysis
Source: Open Med (Wars). 2026 May 7;21(1):20261378. doi: 10.1515/med-2026-1378 (PMC13150366; doi:10.1515/med-2026-1378)
Supplement: Supplementary file 1 — Supplementary Material [file j_med-2026-1378_suppl_001.docx]

Supplementary materials 1: The retrieval strategy of this study.

| **Search strategy in Pubmed** | | |
| --- | --- | --- |
| Search | Query | Results |
| #1 | "Chemoembolization, Therapeutic"[Mesh] | 8,023 |
| #2 | ((Therapeutic Chemoembolization[Title/Abstract]) OR (Chemoembolizations, Therapeutic[Title/Abstract])) OR (Therapeutic Chemoembolizations[Title/Abstract]) | 2,122 |
| #3 | (Transarterial chemoembolization[Title/Abstract]) OR (TACE[Title/Abstract]) | 10,333 |
| #4 | (("Chemoembolization, Therapeutic"[Mesh]) OR (((Therapeutic Chemoembolization[Title/Abstract]) OR (Chemoembolizations, Therapeutic[Title/Abstract])) OR (Therapeutic Chemoembolizations[Title/Abstract]))) OR ((Transarterial chemoembolization[Title/Abstract]) OR (TACE[Title/Abstract])) | 13,593 |
| #5 | ((neutrophil lymphocyte ratio[Title/Abstract]) OR (neutrophil-to-lymphocyte ratio[Title/Abstract])) OR (NLR[Title/Abstract]) | 22,960 |
| #6 | ((("Chemoembolization, Therapeutic"[Mesh]) OR (((Therapeutic Chemoembolization[Title/Abstract]) OR (Chemoembolizations, Therapeutic[Title/Abstract])) OR (Therapeutic Chemoembolizations[Title/Abstract]))) OR ((Transarterial chemoembolization[Title/Abstract]) OR (TACE[Title/Abstract]))) AND (((neutrophil lymphocyte ratio[Title/Abstract]) OR (neutrophil-to-lymphocyte ratio[Title/Abstract])) OR (NLR[Title/Abstract])) | 115 |
| #7 | ((platelet-to-lymphocyte ratio[Title/Abstract]) OR (platelet lymphocyte ratio[Title/Abstract])) OR (plr[Title/Abstract]) | 9,364 |
| #8 | #4 AND #7 | 48 |
|  | **Search strategy in Embase** |  |
| #1 | 'chemoembolization'/exp | 26,047 |
| #2 | 'therapeutic chemoembolization':ti,ab | 1 |
| #3 | 'chemoembolizations, therapeutic':ti,ab | 0 |
| #4 | 'therapeutic chemoembolizations':ti,ab | 0 |
| #5 | 'transarterial chemoembolization':ti,ab | 9,117 |
| #6 | 'tace':ti,ab | 16,091 |
| #7 | #1 OR #2 OR #3 OR #4 OR #5 OR #6 | 31,151 |
| #8 | 'neutrophil lymphocyte ratio':ti,ab | 9,183 |
| #9 | 'neutrophil-to-lymphocyte ratio':ti,ab | 15,168 |
| #10 | 'nlr':ti,ab | 26,944 |
| #11 | #8 OR #9 OR #10 | 32,935 |
| #12 | #7 AND #11 | 260 |
| #113 | 'platelet-to-lymphocyte ratio':ti,ab | 5,443 |
| #14 | 'platelet lymphocyte ratio':ti,ab | 3,091 |
| #15 | 'plr':ti,ab | 11,980 |
| #16 | #13 OR #14 OR #15 | 13,442 |
| #17 | #7 AND #16 | 120 |
|  | **Search strategy in Wed of Science** |  |
| #1 | TS=(Chemoembolization, Therapeutic OR Therapeutic Chemoembolization OR Chemoembolizations, Therapeutic OR Therapeutic Chemoembolizations OR Transarterial chemoembolization OR TACE) | 21,104 |
| #2 | TS=( neutrophil lymphocyte ratio OR neutrophil-to-lymphocyte ratio OR NLR) and Preprint Citation Index | 48,782 |
| #3 | #1 AND #2 | 186 |
| #4 | TS=(platelet-to-lymphocyte ratio OR platelet lymphocyte ratio OR PLR) | 16,479 |
| #5 | #1 AND #4 | 86 |
|  | **Search strategy in Cochrane Library** |  |
| #1 | (Chemoembolization, Therapeutic):ti,ab,kw OR (Therapeutic Chemoembolization):ti,ab,kw OR (Chemoembolizations, Therapeutic):ti,ab,kw OR (Therapeutic Chemoembolizations):ti,ab,kw OR (Transarterial chemoembolization):ti,ab,kw | 1,175 |
| #2 | (TACE):ti,ab,kw | 1,563 |
| #3 | #1 or #2 | 1,885 |
| #4 | (neutrophil lymphocyte ratio):ti,ab,kw OR (neutrophil-to-lymphocyte ratio):ti,ab,kw OR (NLR):ti,ab,kw | 1,615 |
| #5 | #3 and #4 | 2 |
| #6 | (platelet-to-lymphocyte ratio):ti,ab,kw OR (platelet lymphocyte ratio):ti,ab,kw OR (PLR):ti,ab,kw | 752 |
| #7 | #3 and #6 | 0 |

Supplementary materials 2: Quality assessment of included studies by the Newcastle-Ottawa Scale

| Study | Selection | | | | Comparability | Exposure | | | Scores |
| --- | --- | --- | --- | --- | --- | --- | --- | --- | --- |
|  | Adequate definition of cases | Representativeness of the cases | Selection of controls | Definition of controls | Control for important factor | Ascertainment of exposure | Same method of ascertainment for cases and controls | Non-response  rate |  |
| Huang (2011) [16] | ⭐ | ⭐ | ⭐ | ⭐ | ⭐⭐ | ⭐ | ⭐ | ⭐ | 9 |
| Pianto (2012) [17] |  | ⭐ | ⭐ | ⭐ | ⭐ | ⭐ | ⭐ |  | 6 |
| McNally (2013) [18] | ⭐ | ⭐ | ⭐ | ⭐ | ⭐ | ⭐ | ⭐ | ⭐ | 8 |
| Xu (2014) [19] |  | ⭐ | ⭐ | ⭐ | ⭐ | ⭐ | ⭐ | ⭐ | 7 |
| Zhang (2014) [20] | ⭐ |  | ⭐ | ⭐ | ⭐ | ⭐ | ⭐ | ⭐ | 7 |
| Xue (2015) [21] | ⭐ | ⭐ | ⭐ | ⭐ | ⭐⭐ | ⭐ | ⭐ | ⭐ | 9 |
| Tian (2016) [22] | ⭐ |  | ⭐ | ⭐ | ⭐ | ⭐ | ⭐ | ⭐ | 7 |
| Zhou (2016) [23] | ⭐ | ⭐ | ⭐ | ⭐ | ⭐ | ⭐ | ⭐ | ⭐ | 8 |
| Liu1 (2017) [24] |  | ⭐ | ⭐ | ⭐ | ⭐ | ⭐ | ⭐ | ⭐ | 7 |
| Liu2 (2017) [25] | ⭐ | ⭐ | ⭐ | ⭐ | ⭐ | ⭐ | ⭐ | ⭐ | 8 |
| Rebonato (2017) [26] | ⭐ | ⭐ | ⭐ | ⭐ | ⭐⭐ | ⭐ | ⭐ | ⭐ | 9 |
| Chon (2019) [27] | ⭐ | ⭐ | ⭐ | ⭐ | ⭐⭐ | ⭐ | ⭐ | ⭐ | 9 |
| He (2019) [28] | ⭐ | ⭐ | ⭐ | ⭐ | ⭐⭐ | ⭐ | ⭐ | ⭐ | 9 |
| Liu (2020) [29] | ⭐ | ⭐ | ⭐ | ⭐ | ⭐⭐ | ⭐ | ⭐ | ⭐ | 9 |
| Schobert (2020) [8] | ⭐ |  | ⭐ | ⭐ | ⭐ | ⭐ | ⭐ | ⭐ | 7 |
| Wang (2020) [30] | ⭐ | ⭐ | ⭐ | ⭐ | ⭐ | ⭐ | ⭐ | ⭐ | 8 |
| Chu (2021) [31] | ⭐ | ⭐ | ⭐ | ⭐ | ⭐⭐ | ⭐ | ⭐ | ⭐ | 9 |
| Liu (2021) [7] | ⭐ |  | ⭐ | ⭐ | ⭐ | ⭐ | ⭐ | ⭐ | 7 |
| Lu (2021) [12] | ⭐ | ⭐ | ⭐ | ⭐ | ⭐ | ⭐ | ⭐ | ⭐ | 8 |
| Young (2021) [32] | ⭐ | ⭐ | ⭐ | ⭐ | ⭐ | ⭐ | ⭐ | ⭐ | 8 |
| Cho (2022) [33] | ⭐ |  | ⭐ | ⭐ | ⭐ | ⭐ | ⭐ | ⭐ | 7 |
| Wang (2023) [34] | ⭐ | ⭐ | ⭐ | ⭐ | ⭐⭐ | ⭐ | ⭐ | ⭐ | 9 |
| Xi (2023) [35] | ⭐ |  | ⭐ | ⭐ | ⭐ | ⭐ | ⭐ | ⭐ | 7 |
| Zhou (2024) [13] |  | ⭐ | ⭐ | ⭐ | ⭐ | ⭐ | ⭐ |  | 6 |
